# Supplementary material for: Global hypo-methylation in a proportion of glioblastoma enriched for an astrocytic signature is associated with increased invasion and altered immune landscape
Source: eLife. 2022 Nov 22;11:e77335. doi: 10.7554/eLife.77335 (PMC9681209; doi:10.7554/eLife.77335)
Supplement: Figure 2—source data 1. [file elife-77335-fig2-data1.zip › Figure_2_source_data_1/Figure_2I_J/homerResults/motif12.similar.html]

motif12

## Information for motif12

A
G
T
C
A
G
T
C
A
G
C
T
A
G
T
C
C
G
T
A
A
C
T
G
A
G
T
C
A
G
T
C
C
G
T
A
G
A
T
C
A
C
T
G
C
T
A
G
G
T
C
A
A
T
C
G
A
G
T
C
  
Reverse Opposite:  

A
C
T
G
A
T
G
C
A
C
G
T
A
G
T
C
A
G
T
C
C
A
T
G
A
C
G
T
A
C
T
G
A
C
T
G
A
G
T
C
A
C
G
T
A
C
T
G
C
T
G
A
A
C
T
G
A
C
T
G
  

|  |  |
| --- | --- |
| p-value: | 1e-41 |
| log p-value: | -9.624e+01 |
| Information Content per bp: | 1.901 |
| Number of Target Sequences with motif | 47.0 |
| Percentage of Target Sequences with motif | 6.47% |
| Number of Background Sequences with motif | 2.0 |
| Percentage of Background Sequences with motif | 0.70% |
| Average Position of motif in Targets | 93.8 +/- 53.5bp |
| Average Position of motif in Background | 93.6 +/- 4.9bp |
| Strand Bias (log2 ratio + to - strand density) | -0.9 |
| Multiplicity (# of sites on avg that occur together) | 1.13 |
| Motif File: | file (matrix) reverse opposite |

### Similar de novo motifs found

|  |  |  |  |  |  |  |  |
| --- | --- | --- | --- | --- | --- | --- | --- |
| Rank | Match Score | Redundant Motif | P-value | log P-value | % of Targets | % of Background | Motif file |
| 1 | 0.658 | A T C G T G A C G T A C C A T G A T C G G T A C A G C T T A G C T A G C G T C A A C G T A C T G A T C G G A T C G C A T C T A G T C G A C T A G C T A G T C G A T C A G C T A G T A G C C A T G A T C G G A C T A C G T C G A T A T G C C A G T A C G T C A G T A T G C A C T G C T A G | 1e-34 | -78.787631 | 5.65% | 0.48% | motif file (matrix) |
| 2 | 0.752 | A T G C G A T C C T A G A C T G A T G C A G C T A T G C G T A C C T G A A C T G T A C G A T C G T G A C G C A T T A C G C T G A A T C G T C A G C T G A A C T G T A C G T C A G T A C G T A C G T A C G | 1e-32 | -75.877448 | 9.37% | 1.45% | motif file (matrix) |
| 3 | 0.627 | T A C G A G T C A T C G G T A C T A G C C A T G T C A G G A T C A C G T T A G C T G A C T C G A A C G T T A C G T A C G T A G C C G A T T A C G T C G A T C A G T C A G T G C A C A T G C T A G T G A C C A T G A T C G G A C T A C G T C A G T A T G C A C G T A G C T A G C T A G T C A T C G C A T G A C T G A G C T A G C T | 1e-10 | -23.885435 | 3.58% | 0.80% | motif file (matrix) |
